# The mechanism of square dancing on subjective well-being among middle-aged and older women in China: mediating role of social connectedness and moderating role of exercise self-efficacy

**DOI:** 10.3389/fpubh.2025.1701258

**Published:** 2025-11-26

**Authors:** Junmin Wang, Qingzhong Wu, Yongping Xi, Hongtao Xia

**Affiliations:** 1 College of Physical Education, Zhangjiakou University, Zhangjiakou, China; 2 College of Physical Education, Hebei University of Engineering, Handan, China

**Keywords:** square dancing, subjective well-being, social connectedness, exercise self-efficacy, middle-aged and older women

## Abstract

**Objective:**

This study investigates how participation in square dancing influences subjective well-being among middle-aged and older women, focusing on the mediating role of social connectedness and the moderating role of exercise self-efficacy.

**Methods:**

A cross-sectional survey of 365 middle-aged and older women engaged in square dancing was conducted. Validated questionnaires were used to assess square dancing participation, social connectedness, exercise self-efficacy, and subjective well-being. Structural equation modeling and moderated mediation analyses were employed to test the hypothesized model.

**Results:**

Participation in square dancing was positively associated with the subjective well-being of middle-aged and older women (*β* = 0.252, *p* < 0.001), and this association was partially mediated by social connectedness (*β* = 0.175, *p* < 0.001, 40.98% of the total effect). Moreover, exercise self-efficacy moderated both the link between square dancing and social connectedness (*β* = 0.182, *p* < 0.001) and the direct link between square dancing and subjective well-being (*β* = 0.122, *p* < 0.001), indicating a dual moderating role.

**Conclusion:**

Middle-aged and older women who participate in square dancing not only directly enhance their subjective well-being but also indirectly improve it by strengthening their sense of social connectedness. Furthermore, individuals with higher levels of exercise self-efficacy experience more pronounced gains in social connectedness and subjective well-being through square dancing activities.

## 1 Introduction

Since the beginning of the 21st century, Chinese society has been undergoing an accelerated aging process. According to the National Bureau of Statistics, the proportion of the population aged 60 and above reached 22% in 2024, and both the number and proportion of older adults continue to increase (1). In this context, many middle-aged and older adults face multiple challenges, including the “empty-nest” phenomenon, physical decline, and “withdrawal from social roles” (2, 3). These challenges not only weaken social support networks but also reduce subjective well-being. How to improve the quality of life and enhance happiness among older adults in an aging society has thus become an urgent issue in public health and social governance.

Among various leisure-time physical activities for older adults, square dancing is particularly popular among Chinese middle-aged and older women because of its collective nature, accessibility, and cultural significance. It has become an important social practice for promoting active aging in China (4). Prior studies suggest that square dancing not only improves physical health but also fosters social support and psychological satisfaction through group interactions (5, 6). However, the pathways through which square dancing shapes subjective well-being are still not fully clarified, particularly with respect to psychosocial mechanisms and potential moderating influences.

In response to this research gap, the present study develops a moderated mediation framework to examine how participation in square dancing influences subjective well-being through social connectedness and exercise self-efficacy. Although previous studies have explored the health benefits of physical activity, few have specifically investigated the social and psychological mechanisms through which square dancing, as a community-based group exercise, promotes well-being among middle-aged and older women. This study aims to address this gap and enrich evidence on the psychosocial pathways linking physical activity to well-being.

## 2 Literature review and hypotheses

### 2.1 Square dancing and subjective well-being

Subjective well-being (SWB) is a key indicator of an individual’s quality of life and mental health, generally encompassing three dimensions: life satisfaction, the presence of positive emotions, and the absence of negative emotions (7). With the rapid acceleration of population aging, enhancing the subjective well-being of middle-aged and older adults has become a central concern in both public health and social policy. Prior studies consistently demonstrate that active leisure and sports activities are vital for improving mental health and increasing happiness among middle-aged and older women (8, 9).

In the Chinese context, square dancing—characterized by its collective nature, low cost, and accessibility—has attracted growing academic attention in recent years. Evidence shows that participation in square dancing not only improves physical health but also regulates emotions through the combined effects of rhythmic music and physical movement, thereby enhancing positive affective experiences (10). Moreover, because square dancing is typically performed in groups, it serves as a key venue for social interaction and emotional support, further contributing to greater life satisfaction and happiness (11). Therefore, square dancing is not only a form of physical activity but also a powerful means of promoting emotional regulation and enhancing well-being.

*H1:* Square dancing is positively associated with the subjective well-being of middle-aged and older women.

### 2.2 The mediating role of social connectedness

Social connectedness refers to an individual’s perceived sense of belonging, acceptance, and the quality of interpersonal interactions within social relationships. It is widely recognized as a crucial psychosocial factor influencing subjective well-being (12). Numerous studies indicate that positive social relationships can alleviate stress, enhance well-being, and reduce feelings of loneliness and depression (13, 14). Consequently, social connectedness is widely recognized as a key mechanism for promoting mental health and overall well-being.

In the field of sports, growing evidence suggests that the impact of group activities on well-being extends beyond physical health improvements to include enhanced social connections among participants. For instance, Stevens et al. (15) found that engaging in group sports significantly boosts mental health and life satisfaction by strengthening feelings of belonging and social support. Similarly, the “social therapy” theory proposed by Cruwys et al. (16) suggests that group activities elevate well-being by strengthening social recognition and interpersonal bonds. Collectively, as a group-oriented activity, square dancing may exert a dual effect on subjective well-being: directly fostering social connections and indirectly strengthening social bonds. Beyond the general framework of social connectedness, the social identity approach to health (17) provides a complementary perspective for understanding how group-based activities promote well-being. According to this theory, identification with a social group fosters psychological safety, collective efficacy, and a sense of belonging, which together serve as “social cures” that protect mental health. In the context of square dancing, participants’ shared identity and mutual recognition as group members may enhance emotional security and social trust, reinforcing both social connectedness and subjective well-being in aging populations.

*H2:* Social connectedness mediates the relationship between square dancing and subjective well-being.

### 2.3 The moderation of exercise self-efficacy

Exercise Self-Efficacy (ESE) refers to an individual’s confidence in their ability to persist in physical exercise and achieve predetermined goals despite obstacles or difficulties (18). Social cognitive theory posits that self-efficacy is a key psychological resource shaping individual behavior. It not only determines whether people will actively engage in specific behaviors but also influences the persistence and ultimate effectiveness of those behaviors (19). In the field of physical education and health, extensive research confirms a significant positive correlation between exercise self-efficacy and levels of physical activity participation, psychological well-being, and life satisfaction (20, 21).

Previous research has emphasized that exercise self-efficacy plays a crucial role in linking physical activity to psychological outcomes. Individuals with higher exercise self-efficacy are better equipped to cope with difficulties and obstacles encountered during physical activity, which helps sustain engagement and motivation (22). Furthermore, exercise self-efficacy is associated with stronger social motivation and a greater willingness to interact with others (23). Thus, those with higher self-efficacy are more likely to translate exercise participation into positive social relationships, thereby enhancing their perceived sense of social connectedness.

For many middle-aged and older women, decreased physical function, chronic disease burdens, and psychological difficulties may reduce their confidence and perseverance in exercise. However, empirical studies have shown that those with higher levels of exercise self-efficacy are more likely to actively participate in group activities, obtain more social support and emotional connections, and consequently experience improved mental health and well-being (24, 25). Therefore, exercise self-efficacy is not only a key psychological factor influencing participation in physical activity among middle-aged and older women, but it may also serve as a moderating variable that conditions how square dancing contributes to subjective well-being through social connectedness.

*H3:* Exercise self-efficacy moderates the relationship between square dancing and social connectedness.

*H4:* Exercise self-efficacy moderates the relationship between square dancing and subjective well-being.

### 2.4 Conceptual model and hypotheses

**Figure 1:**
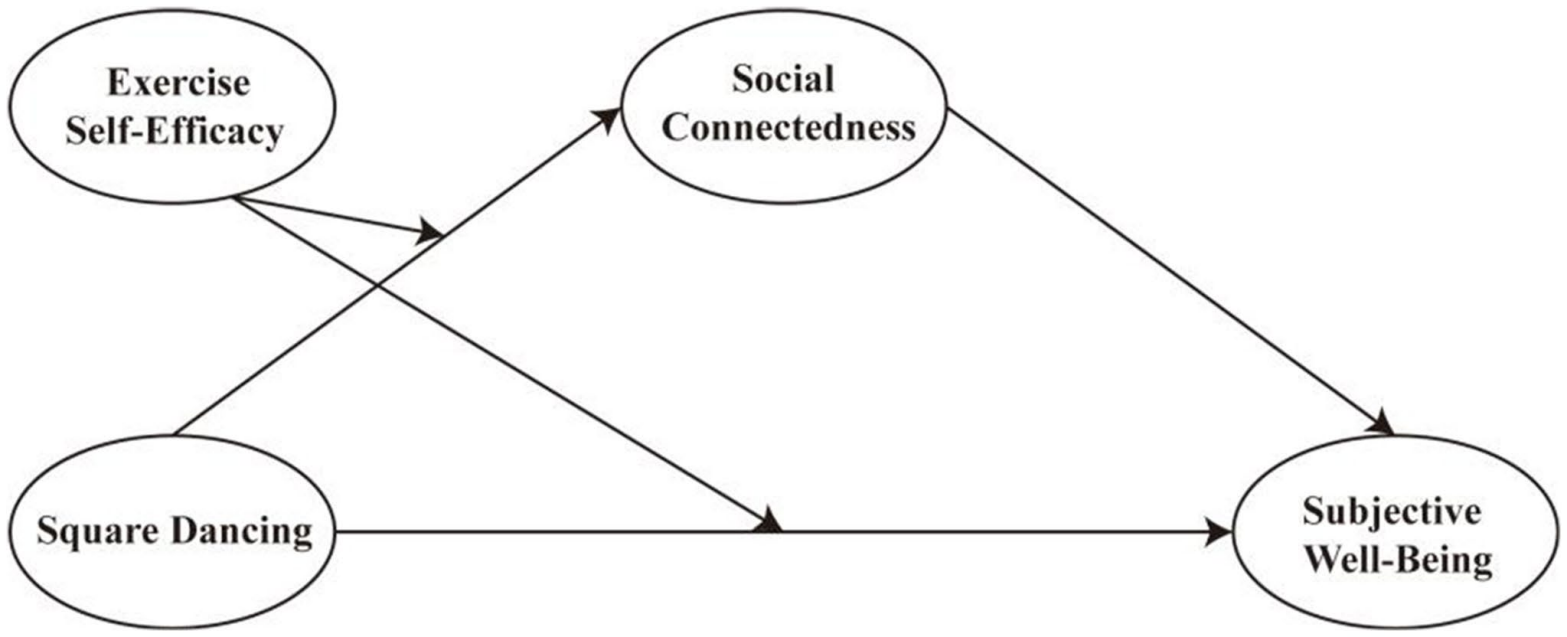
Conceptual model of the moderated mediation framework.

Drawing on the preceding discussion, this research develops a moderated mediation framework (Figure 1). In this model, participation in square dancing is expected to be positively associated with the subjective well-being of middle-aged and older women both directly and indirectly through social connectedness. Moreover, exercise self-efficacy is conceptualized as a moderating factor that may condition the strength of these associations, shaping how square dancing relates to social connectedness and subjective well-being.

## 3 Materials and methods

### 3.1 Participants and procedure

This study focused on middle-aged and older women who regularly participated in square dancing. From April to June 2024, the research team recruited participants from several community square dance groups in Chengdu, Sichuan Province. Using a combination of convenience and snowball sampling, a total of 365 valid questionnaires were collected. A cross-sectional design was adopted as a practical and ethical approach to examine the associations among square dancing, social connectedness, and subjective well-being in natural community settings, allowing for efficient data collection from a large group of participants within a limited time frame.

Participants were eligible if they (a) were female and aged 45 years or older, (b) had regularly participated in square dancing for at least six consecutive months, and (c) reported no serious cardiovascular, cognitive, or musculoskeletal disorders that might restrict participation. Those with severe mobility limitations or chronic illnesses requiring long-term medication adjustments were excluded.

Data were collected in collaboration with nine community cultural and sports organizations, covering twelve distinct square dance troupes located in both urban and suburban areas of Chengdu. The typical group size ranged from 25 to 40 members, and they generally practiced 4–6 times per week for about 1–1.5 h per session. Investigators visited each group site to distribute and collect questionnaires before or after dance sessions. To minimize potential clustering effects from participants belonging to the same troupe, troupe identifiers were recorded, and robust standard errors clustered by troupe were computed in supplementary analyses to verify robustness.

During data collection, trained investigators worked closely with group leaders to ensure voluntary participation. On-site guidance was provided to clarify questionnaire items and ensure independent completion. For respondents with limited literacy, oral responses were recorded verbatim by investigators to prevent misinterpretation. Of the 380 questionnaires distributed, 365 valid responses were obtained (response rate = 96.1%). Cases with excessive missing values (>10% unanswered items) were excluded, and no significant differences were found between included and excluded cases in terms of age or dance experience.

All participants took part voluntarily and provided written informed consent after being briefed on the study’s objectives, data confidentiality, and anonymity principles. For those with limited literacy, investigators read the consent statement aloud and documented verbal consent. The study was conducted in accordance with the Declaration of Helsinki and approved by the Ethics Committee of Zhangjiakou University (Approval No. 2025–24, April, 2025). All data were anonymized and used exclusively for academic purposes.

### 3.2 Instruments

#### 3.2.1 Square dancing

This study referred to the classic framework for measuring physical activity internationally (26) and combined it with the commonly used “frequency, duration and organization” dimension in group sports research (27). Prior research in community and development sport highlights that the degree of organization in group activities critically shapes participants’ social and psychological outcomes. Structured groups with clear roles and cooperative goals enhance members’ sense of belonging and commitment (28, 29). Accordingly, the “degree of organization” in this study reflects how structured and cohesive square dance groups are. In the context of China, empirical studies have shown that these dimensions can effectively predict the physical health, social support, and happiness of middle-aged and older people (10, 30, 31). Therefore, based on the above research, this study divides the measurement of participation in square dancing into four dimensions: participation frequency, participation duration, participation years, and degree of organization. All items are scored using the five-point Likert scale, with higher scores indicating higher participation in square dancing.

#### 3.2.2 Social connectedness

This study employed the Social Connectedness Scale (SCS) developed by Lee and Robbins to assess social connectedness. This scale encompasses multiple dimensions including a sense of belonging, intimacy, and acceptance, and has been widely applied in previous research (12). Subsequent studies simplified or adapted the scale for group sports and recreational activity settings, confirming its applicability among middle-aged and older adults (32). Building upon existing research, this study employs a multidimensional approach to measure social connectedness, focusing specifically on the dimensions of acceptance, belonging, and intimacy within interpersonal relationships. All items were rated on a five-point Likert scale (1 = Strongly Disagree, 5 = Strongly Agree), with higher scores indicating greater social connectedness.

#### 3.2.3 Exercise self-efficacy

This study used the Self Efficacy for Exercise Scale (SEE) developed by Resnick and Jenkins (33), which has been widely validated among middle-aged and older population and has good reliability and validity (33, 34). The core dimension of this scale assesses individuals’ confidence in maintaining exercise despite fatigue, inclement weather, busy schedules, or physical discomfort. To maintain consistency across all instruments and simplify response choices for older participants, the SEE was administered using this five-point format, which demonstrated good measurement reliability and validity in the present study. Higher scores indicate stronger exercise self-efficacy.

#### 3.2.4 Subjective well-being

Subjective well-being was assessed using two complementary scales to capture both its cognitive and affective dimensions. The cognitive aspect was measured by the Satisfaction With Life Scale (SWLS) developed by Diener et al. (35), which includes five items rated on a five-point Likert scale (1 = Strongly disagree, 5 = Strongly agree); higher scores indicate greater life satisfaction. The affective aspect was measured by the World Health Organization Five Well-Being Index (WHO-5) (36). To ensure consistency with the SWLS and maintain the conceptual coherence of the overall subjective well-being construct, the WHO-5 items were assessed using a five-point response format (1 = At no time, 5 = All of the time). This scoring approach provided a unified range across instruments and facilitated the integration of cognitive and affective indicators within a single latent construct. Both scales demonstrated good internal reliability in the present sample (37, 38).

### 3.3 Control variables

To minimize the interference of confounding factors on the research findings, this study incorporated several control variables into the model analysis. Based on existing literature and the characteristics of the middle-aged and older adults, age, marital status, educational attainment, and monthly income were selected as control variables (39). Research has shown that these variables are potentially associated with subjective well-being and exercise participation. Accordingly, they were incorporated into the regression and structural equation modeling analyses.

### 3.4 Statistical analyses

The statistical analyses were carried out with SPSS 26.0, AMOS 24.0, and Hayes’ PROCESS macro (v3.4). To assess potential common method bias (CMB), the Common Latent Factor (CLF) approach was employed. A latent method factor was added to the confirmatory factor analysis (CFA) model, allowing all observed indicators to load simultaneously on both their theoretical constructs and the CLF. Following the recommendations of Podsakoff et al. (40), model fit indices were compared between the original and the CLF-adjusted models. If the changes in GFI, CFI, TLI, and RMSEA indices are less than 0.01, then CMB is not considered a serious issue, CMB is considered not a serious concern (41). In this study, the differences in model fit indices were minimal (ΔGFI < 0.01, ΔCFI < 0.01, ΔTLI < 0.01, ΔRMSEA < 0.01), suggesting that common method bias was not a major issue.

Descriptive statistics, reliability assessments, and Pearson correlations were first calculated in SPSS to examine distributional characteristics and internal consistency. Subsequently, confirmatory factor analyses (CFAs) were conducted in AMOS to verify the measurement validity of all constructs. Before conducting the CFA, all subjective well-being items (SWLS and WHO-5) were coded on the same 1–5 response scale and oriented in the same positive direction, ensuring consistent measurement prior to model estimation. The results demonstrated satisfactory reliability, convergent validity, and discriminant validity. After validating the measurement model, composite mean scores of all latent constructs were computed for subsequent analyses.

Mediation and moderation effects were then examined using Hayes’ PROCESS macro (Model 8) with 5,000 bootstrap samples to generate bias-corrected confidence intervals. All continuous variables were mean-centered before creating interaction terms, and heteroskedasticity-consistent standard errors (HC3) were used. Adding control variables to the model equation does not change the mode or significance of the main coefficients. Therefore, for the simplicity and clarity of the model, the final report model is estimated without control variables. For significant interaction effects, simple slope analyses and Johnson–Neyman tests were employed to probe the conditional effects at low (−1 SD), mean, and high (+1 SD) levels of the moderator.

## 4 Results

### 4.1 Descriptive statistics and correlations among the main study variables

As shown in Table 1, a total of 365 valid participants were included in this study. The majority were aged between 45 and 69 years (90.96%), and most were married (94.25%). In terms of education, 16.16% had primary school or below, while the largest groups had junior high school (34.25%) or high school education (36.44%), and 13.15% had college or above. Monthly income was mainly concentrated between RMB 1,001 and 3,000 (67.95%). Overall, the sample was characterized by middle-aged and older women with stable marital status, moderate education levels, and low-to-middle income.

**Table 1:** Demographic characteristics of the samples (*N* = 365).

Variable	Category	Frequency (*n*)	Percentage (%)
Age	45–54	154	42.19%
	55–69	178	48.77%
	70–79	24	6.57%
	>80	9	2.47%
Marital status	Married	344	94.25%
	Divorced/Single	12	3.29%
	Widowed	9	2.46%
Education level	Primary school or below	59	16.16%
	Junior high school	125	34.25%
	High school	133	36.44%
	College or above	48	13.15%
Monthly income(RMB)	<1,000	94	25.75%
	1,001-3,000	248	67.95%
	3,001-5,000	21	5.75%
	>5,000	2	0.55%

Table 2 presents the means, standard deviations, and Pearson correlations for the main study variables. The mean scores ranged from 2.95 to 3.01, and the standard deviations (0.84–1.05) indicated moderate variability. All variables were significantly correlated in the expected theoretical direction. Furthermore, none of the correlations exceeded the commonly accepted multicollinearity threshold of 0.85, and all variance inflation factor (VIF) values were within acceptable limits, suggesting that multicollinearity was not a concern.

**Table 2:** Descriptive statistics and correlations among primary variables.

Variable	M	SD	1	2	3	4
1. Square dancing	2.97	0.98	1			
2. Social connectedness	2.95	1.05	0.548^**^	1		
3. Exercise self-efficacy	3.01	1.04	0.250^**^	0.276^**^	1	
4. Subjective well-being	3.01	0.84	0.500^**^	0.535^**^	0.157^**^	1

*p < 0.05, **p < 0.01 (two-tailed).

### 4.2 The test of reliability and validity

Table 3 presents the results of the reliability and convergent validity analysis for the measurement model. Each construct includes multiple observed items, with their standardized factor loadings, Cronbach’s *α* coefficients, composite reliability (CR), and average variance extracted (AVE) reported. All standardized loadings exceed 0.65, indicating adequate item reliability. The Cronbach’s α coefficients for the four latent constructs range from 0.869 to 0.917, demonstrating high internal consistency reliability. The CR values (ranging from 0.869 to 0.926) and AVE values (ranging from 0.525 to 0.559) also meet the recommended thresholds of 0.70 and 0.50, respectively (42). These results confirm that the measurement model possesses satisfactory reliability and convergent validity.

**Table 3:** Validity and reliability tests of the questionnaires.

Construct	Item	Standardized loading	Cronbach’s *α*	CR	AVE
Square dancing	I participated in square dancing on more days per week on average during the past week.	0.718	0.897	0.899	0.529
	I engaged in square dancing very frequently during the past week.	0.696			
	Each of my square dance sessions usually lasts more than 30 min.	0.690			
	I am able to maintain participation in square dancing for a relatively long duration each time.	0.767			
	I have been participating in square dancing for many years.	0.823			
	Square dancing has become a long-term leisure activity that I have consistently practiced.	0.734			
	I belong to a regular square dance team and often participate in activities with this team.	0.666			
	I have taken on a specific role in my square dance team (e.g., lead dancer, organizer, or active participant).	0.716			
Social connectedness	I feel accepted and recognized by others in my square dance team.	0.694	0.870	0.869	0.527
	I feel that I am regarded as a valued member of the team.	0.732			
	I consider myself an indispensable part of the square dance team.	0.707			
	Participating in square dancing gives me a strong sense of group belonging.	0.731			
	I have developed close relationships with members of my square dance team.	0.754			
	I have friends in the team whom I can confide in and rely on.	0.737			
Exercise self-efficacy	Even when I feel tired, I am confident that I can keep participating in square dancing.	0.758	0.869	0.869	0.525
	Even when the weather is bad, I am confident that I can keep participating.	0.750			
	Even when my schedule is busy, I can still find time to participate.	0.705			
	Even when I feel slightly unwell, I am confident that I can continue participating.	0.677			
	Even when my friends or family do not accompany me, I can still keep participating.	0.727			
	Even when faced with new or difficult dance moves, I am confident that I can master them through practice.	0.729			
Subjective well-being	In most ways, my life is close to my ideal.	0.703	0.917	0.926	0.559
	The conditions of my life are excellent.	0.747			
	I am satisfied with my life.	0.682			
	So far, I have gotten the important things I want in life.	0.721			
	If I could live my life over, I would change almost nothing.	0.708			
	I have felt cheerful and in good spirits.	0.732			
	I have felt calm and relaxed.	0.752			
	I have felt active and vigorous.	0.688			
	I woke up feeling fresh and rested.	0.733			
	My daily life has been filled with things that interest me.	0.726			

CR, composite reliability; AVE, average variance extracted.

In addition, multiple fit indices were used to evaluate the overall goodness of fit of the measurement model, including χ^2^, χ^2^/df, GFI, CFI, TLI, SRMR, and RMSEA. As shown in Table 4, the model demonstrated excellent fit to the data (χ^2^ = 451.274, χ^2^/df = 1.131). All incremental fit indices exceeded the recommended threshold of 0.90 (GFI = 0.927, CFI = 0.991, TLI = 0.990), indicating strong model fit. Moreover, the SRMR value (0.035) and RMSEA value [0.019, 90% CI (0.01, 0.03)] were well below the conventional cut-off of 0.08, suggesting a close and acceptable fit between the hypothesized model and the observed data (43).

**Table 4:** Model fit indices for the measurement model.

Fit indices	χ^2^	χ^2^/df	GFI	CFI	TLI	SRMR	RMSEA/90%CI
Indices	451.274	1.131	0.927	0.991	0.990	0.035	0.019 [0.01, 0.03]

GFI, goodness of fit index; CFI, comparative fit index; TLI, Tucker–Lewis index; SRMR, standardized root mean square residual; RMSEA, root mean square error of approximation.

### 4.3 The mediation model analysis

To test the mediating role of social connectedness in the relationship between square dancing and subjective well-being, regression based mediation analysis was conducted using the PROCESS macro (Model 4), and 5,000 guided resamples were performed. Control variables (age, marital status, education, and monthly income) were initially entered into the models but were found non-significant; for parsimony, they were excluded from the final models, with detailed outputs presented in the Supplementary material.

As shown in Table 5, square dancing significantly positively predicted social connectedness (*β* = 0.585, *p* < 0.001). In the subsequent regression, square dancing (*β* = 0.252, *p* < 0.001) and social connectedness (*β* = 0.300, *p* < 0.001) both significantly and positively predicted subjective well-being, indicating the presence of potential mediating effects.

**Table 5:** Regression analysis of the mediation model.

Predictor	Step 1 (Social connectedness)			Step 2 (Subjective well-being)		
	*β*	SE	t	*β*	SE	t
Square dancing	0.585	0.045	12.945^***^	0.252	0.046	5.491^***^
Social connectedness				0.300	0.042	7.176^***^
*R* ^2^	0.301			0.347		
*F*	167.575^***^			104.700^***^		

*p < 0.05, **p < 0.01, and ***p < 0.001.

Table 6 shows the bootstrap results. The overall impact of square dancing on subjective well-being is significant [*β* = 0.427, 95% confidence interval (0.350, 0.505)]. The direct effect remains significant [*β* = 0.252, 95% confidence interval (0.162, 0.343)], accounting for 59.02% of the total effect, thus supporting H1. The indirect effect generated by social connectedness is also significant [*β* = 0.175, 95% confidence interval (0.124, 0.230)], explaining 40.98% of the total effect and supporting H2. These findings provide empirical evidence for some mediation models, indicating that square dancing directly and indirectly improves subjective well-being by enhancing social connectedness.

**Table 6:** Bootstrapping results of the mediation model.

Effect type	Effect	SE	95% CI	Ratio to total effect
Direct effect	0.252	0.046	[0.162, 0.343]	59.02%
Indirect effect	0.175	0.027	[0.124, 0.230]	40.98%
Total effect	0.427	0.039	[0.350, 0.505]	-

### 4.4 The moderation model analysis

**Figure 2:**
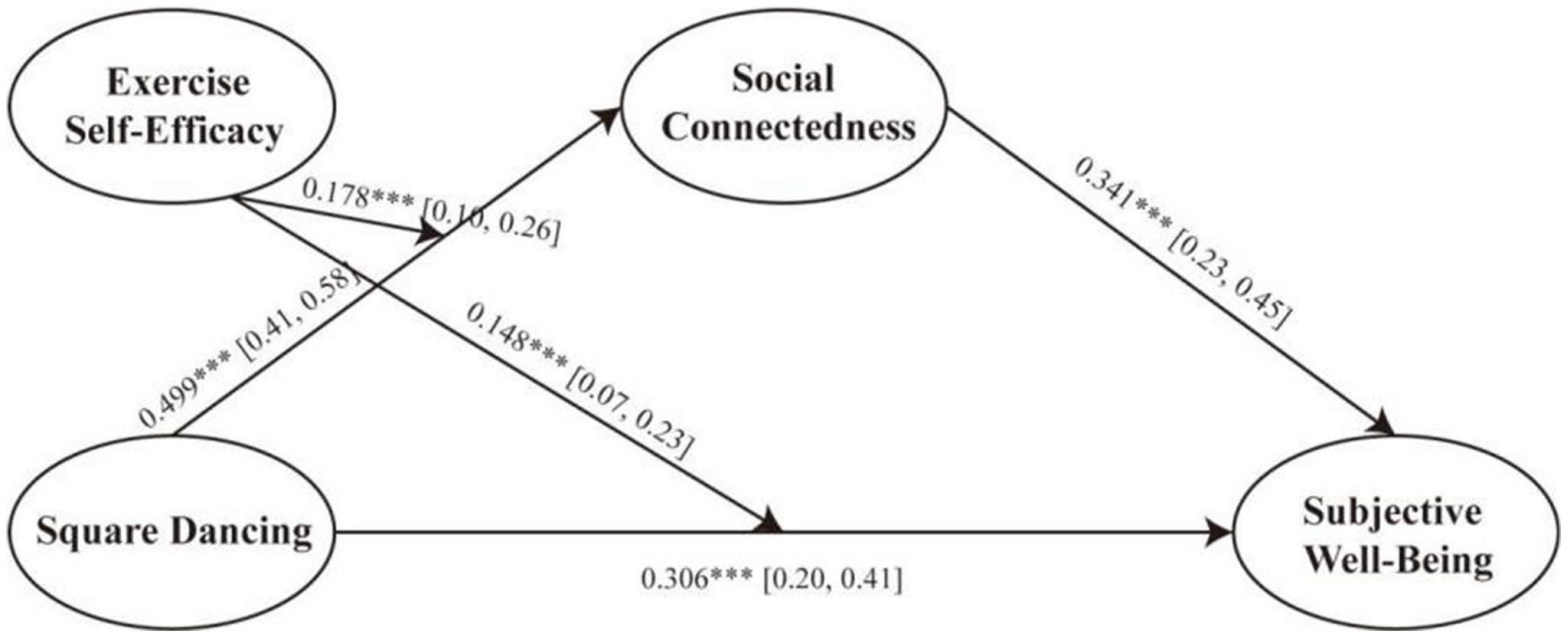
Final moderated mediation model. Values are standardized path coefficients (*β*) obtained from PROCESS Model 8 with 5,000 bootstrap samples. Brackets indicate 95% bias-corrected confidence intervals (CI). ***p < 0.001.

The moderating role of exercise self-efficacy (ESE) in the associations between square dancing participation (SDP), social connectedness, and subjective well-being was examined using PROCESS Model 8. The final moderation-mediation model path diagram is shown in Figure 2.

As shown in Table 7, the interaction between square dancing and exercise self-efficacy was significant for both social connectedness [*β* = 0.182, *p* < 0.001, 95% CI (0.10, 0.27)] and subjective well-being [*β* = 0.122, *p* < 0.001, 95% CI (0.05, 0.19)].

**Table 7:** Moderating effects of exercise self-efficacy on the relationships between square dancing, social connectedness, and subjective well-being.

Dependent variable	*β*	*t*	*p*	95% CI (LLCI, ULCI)	Conditional effects of SDP at levels of ESE	*b*	*p*	95% CI (LLCI, ULCI)	J–N threshold
Social connectedness	0.182	4.129	<0.001	[0.10, 0.27]	Low (−1 SD)	0.34	<0.001	[0.21, 0.48]	−1.86
					Mean	0.53	<0.001	[0.43, 0.62]	
					High (+1 SD)	0.72	<0.001	[0.60, 0.85]	
Subjective well-being	0.122	3.398	<0.001	[0.05, 0.19]	Low (−1 SD)	0.14	0.015	[0.03, 0.25]	−1.19
					Mean	0.26	<0.001	[0.18, 0.35]	
					High (+1 SD)	0.39	<0.001	[0.27, 0.50]	

Simple slope analyses further revealed that the positive effect of square dancing on social connectedness increased with higher levels of exercise self-efficacy: *b* = 0.34 [*p* < 0.001, 95% CI (0.21, 0.48)] at low (−1 SD), *b* = 0.53 [*p* < 0.001, 95% CI (0.43, 0.62)] at mean, and *b* = 0.72 [*p* < 0.001, 95% CI (0.60, 0.85)] at high (+1 SD) levels of self-efficacy. The Johnson–Neyman analysis identified −1.86 (standardized value) as the threshold above which the effect of square dancing on social connectedness became significant.

Similarly, when subjective well-being served as the dependent variable, the positive effect of square dancing also strengthened as self-efficacy increased: *b* = 0.14 [*p* = 0.015, 95% CI (0.03, 0.25)] at low (−1 SD), *b* = 0.26 [*p* < 0.001, 95% CI (0.18, 0.35)] at mean, and *b* = 0.39 [*p* < 0.001, 95% CI (0.27, 0.50)] at high (+1 SD) levels. The Johnson–Neyman significance region began at −1.19, indicating that the moderating effect was significant.

These findings demonstrate that individuals with higher exercise self-efficacy gain stronger social and emotional benefits from square dancing participation, supporting Hypotheses 3 and 4.

**Figure 3:**
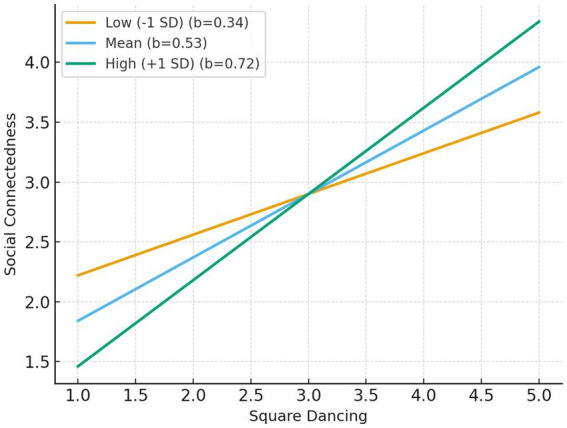
Interaction effect of square dancing and exercise self-efficacy on social connectedness.

**Figure 4:**
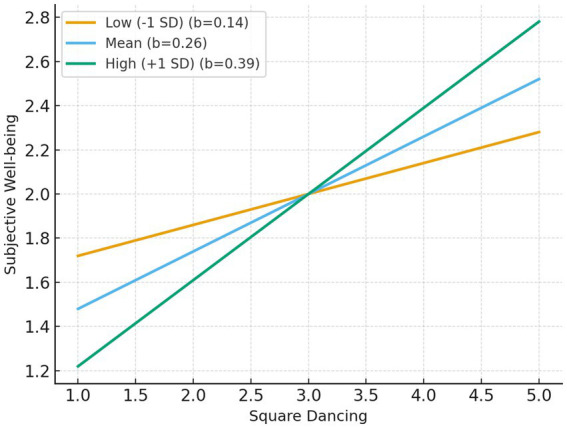
Interaction effect of square dancing and exercise self-efficacy on subjective well-being.

Figures 3, 4 illustrate the moderating role of exercise self-efficacy in the relationship between square dancing and both social connectedness and subjective well-being. As shown, the positive association between square dancing and social connectedness (Figure 3) was stronger among individuals with higher exercise self-efficacy, whereas the slope was relatively weaker for those with lower self-efficacy. A similar pattern emerged for subjective well-being (Figure 4): individuals with higher exercise self-efficacy experienced greater gains in well-being as their participation in square dancing increased. These interaction patterns confirm that exercise self-efficacy enhances the beneficial psychological effects of square dancing participation.

In addition, the conditional indirect effects of square dancing on subjective well-being through social connectedness were examined at different levels of exercise self-efficacy (Table 8). The results indicated that the indirect effect was significant across all levels of exercise self-efficacy, but increased progressively from low [−1 SD, *b* = 0.094, 95% CI (0.049, 0.142)] to mean [*b* = 0.145, 95% CI (0.097, 0.195)] and high levels [+1 SD, *b* = 0.197, 95% CI (0.134, 0.260)]. This pattern further suggests that individuals with higher exercise self-efficacy not only experienced stronger direct effects of square dancing on well-being but also benefited more from the social connectedness pathway.

**Table 8:** Conditional effect analysis at values of exercise self-efficacy.

Conditional direct effect analysis at values of exercise self-efficacy (M ± SD)			
Level of ESE	Direct Effect (b)	BootSE	95% CI (LLCI, ULCI)
Low (−1 SD)	0.136	0.056	[0.026, 0.245]
Mean	0.262	0.043	[0.177, 0.348]
High (+1 SD)	0.389	0.059	[0.274, 0.505]

Conditional indirect effect analysis at values of exercise self-efficacy (M ± SD)			
Level of ESE	Indirect Effect (b)	BootSE	95% CI (LLCI, ULCI)
Low (−1 SD)	0.094	0.025	[0.050, 0.145]
Mean	0.145	0.026	[0.098, 0.198]
High (+1 SD)	0.197	0.033	[0.134, 0.264]

## 5 Discussion

### 5.1 The direct effect of square dancing on subjective well-being

The results of this study indicate that square dancing is positively associated with the subjective well-being of middle-aged and older women. This association is first reflected at the physical level: subjective well-being is often linked to individuals’ positive perceptions of their own physiological state (44). The rhythmic and regular movement patterns characteristic of square dancing are associated with better perceived physical function and body image among middle-aged and older women, which in turn correlate with higher levels of perceived health and self-evaluation. Secondly, compared to individualized forms of movement, square dancing has distinct group and interactive characteristics, and its “collective atmosphere infection” effect plays an important role in emotional regulation. Specifically, cheerful music rhythms, uniform movements, and positive interactions between groups can create a strong emotional resonance atmosphere, making it easier for individuals to experience positive emotions such as happiness and relaxation during exercise (45). Positive emotions not only directly enhance happiness, but also further increase individuals’ engagement in sports, forming a positive cycle of exercise and emotional engagement. From a social-cognitive perspective, such positive emotional experiences may enhance individuals’ perceived competence and self-efficacy, which in turn reinforce motivation and participation behaviors.

### 5.2 The mediating role of social connectedness

This study further confirms that social connectedness plays a mediating role in the impact of square dancing on the subjective well-being of middle-aged and older women. According to social support theory, stable social connections and emotional support are important external supports for subjective well-being (46). Square dancing, with its unique “group participation” feature, provides a scene carrier for middle-aged and older women to continuously build social networks. Compared with intergenerational interactions within families or occasional interactions between neighbors, square dancing has the triple stability of “fixed time, fixed place, and fixed group,” allowing participants to form a “like-minded social circle” based on common interests through high-frequency repetitive interactions.

In the specific process, the coordination of movements and mutual guidance during exercise constitute instrumental support, while the sharing of experiences and emotional resonance during rest and communication form emotional support (47). The two together form a dual support system of “tools and emotions,” effectively alleviating the “social alienation” and “role loss” felt by middle-aged and older women due to their children’s independence and retirement role changes. In addition, there is a significant “peer effect” in square dance groups, such as completing performance tasks together, encouraging each other to persist in sports, and this collective experience can strengthen individuals’ “sense of belonging” and “identity” (48, 49). The satisfaction of belonging is not only an important manifestation of social connectedness, but also a key source of subjective well-being emotional experience dimension (50). Therefore, the sense of social connection becomes an important intermediary link for square dancing to promote subjective well-being through the mechanism of “social support and sense of belonging satisfaction.”

### 5.3 The moderating effect of exercise self-efficacy

Guided by social-cognitive theory, the findings of this study indicate that exercise self-efficacy moderates the associations between square dancing and subjective well-being through two distinct pathways. Specifically, self-efficacy strengthens the positive associations between square dancing and social connectedness and intensifies the overall relationship with subjective well-being. Thus, the observed links between square dancing and well-being vary depending on individual differences in self-efficacy levels.

According to social cognitive theory, self-efficacy represents an individual’s belief in their ability to accomplish tasks and is a key determinant of motivation, persistence, and social behavior (19). Individuals with higher self-efficacy tend to set positive goals and maintain sustained engagement in square dancing, which is associated with a stronger sense of control and achievement. In turn, these beliefs are related to greater perceived social connectedness and higher levels of subjective well-being (10, 51).

In contrast, women with lower self-efficacy often show a tendency toward social avoidance when participating in square dancing, which is associated with lower levels of interaction and engagement. Those with higher self-efficacy, however, tend to adopt more proactive social strategies—such as mentoring peers or assuming leadership roles—which are linked to greater recognition, a stronger sense of belonging, and higher perceived group identity. Collectively, these patterns suggest that self-efficacy is associated with variations in the degree of social connectedness individuals experience in group activities.

Beyond its indirect association, self-efficacy also appears to strengthen the direct relationship between square dancing and well-being. Women with higher self-efficacy tend to report greater physical enjoyment and psychological satisfaction, and they often perceive these positive experiences as related to their own capabilities, which correlate with higher levels of life satisfaction and emotional well-being (52). Taken together, self-efficacy shows a dual moderating pattern, being positively associated with both the link between square dancing and social connectedness and the link between square dancing and subjective well-being.

### 5.4 Implications

This study yields several important implications. Theoretically, this study extends social-cognitive theory to the context of community-based physical activity by identifying how exercise self-efficacy functions as a key boundary condition linking behavioral engagement and psychological outcomes. It also highlights social connectedness as a contextual factor through which social-cognitive processes translate into perceived well-being. It highlights the value of examining culturally embedded, community-based group exercise when considering pathways to active aging and psychological health.

Practically, the findings suggest that square dancing can serve as an effective and low-cost strategy for promoting well-being among middle-aged and older women. Community organizers and policymakers should provide adequate public spaces and institutional support to encourage wider participation. Moreover, interventions aimed at enhancing exercise self-efficacy—through goal setting, positive feedback, and peer modeling—may amplify both the social and psychological benefits of square dancing. Finally, as a culturally distinctive form of collective leisure activity, square dancing offers insights for the design of community-based exercise programs in other cultural contexts seeking to foster social integration and healthy aging.

### 5.5 Limitations

Although this study has certain theoretical and practical value in revealing the mechanisms through which square dancing relates to the subjective well-being of middle-aged and older women, several limitations should be acknowledged.

First, the cross-sectional and self-reported design limits causal inference and may be subject to common method and social desirability biases. Although statistical remedies were applied, these concerns cannot be entirely eliminated. Future longitudinal or experimental designs could better establish temporal and causal relationships.

Second, participants were recruited from specific community dance groups in limited regions, which constrains the generalizability of the findings. Future studies should include more diverse samples and conduct cross-cultural comparisons to enhance external validity.

Third, the current model focused on the mediating role of social connectedness and the moderating role of exercise self-efficacy, while other potentially relevant factors—such as social network size, group cohesion, and physical health—were not considered. Future studies could include these additional variables or test theoretically grounded alternative models, such as conceptualizing self-efficacy as a mediator (square dancing → self-efficacy → social connectedness) or specifying social connectedness as a moderator (individuals with stronger pre-existing social ties may derive greater psychological benefits from group activity). These robustness checks would strengthen the theoretical consistency and explanatory power of the framework.

Fourth, the public nature of square dancing may simultaneously foster belongingness and induce social evaluation pressure. This “double-edged” visibility effect warrants further investigation as a potential boundary condition.

Finally, certain measurement adaptations should be noted. The Self-Efficacy for Exercise (SEE) scale was administered using a five-point Likert format instead of the original 0–10 confidence scale, and both the SWLS and WHO-5 were implemented using a unified five-point format to ensure consistency and reduce respondent burden. Although the current CFA supported acceptable structural validity, future studies should further confirm the psychometric equivalence of these adapted scales and examine age-related differences, particularly among women aged 70 and above.

## 6 Conclusion

This study validated the interaction between square dancing, social connectedness, subjective well-being, and exercise self-efficacy by constructing a moderated mediation model. The research results indicate that square dancing not only has a significant direct promoting effect on the subjective well-being of middle-aged and older women, but also indirectly affects their subjective well-being by enhancing their sense of social connection; Meanwhile, exercising self-efficacy played a crucial regulatory role in the model, enhancing the positive effects of square dancing on social connectedness and subjective well-being. This discovery reveals the unique value of square dancing in promoting mental health and happiness among middle-aged and older people, and emphasizes the importance of enhancing individual exercise self-efficacy.

## Data Availability

The original contributions presented in the study are included in the article/Supplementary material, further inquiries can be directed to the corresponding author.
