# Supplementary material for: The mechanism of square dancing on subjective well-being among middle-aged and older women in China: mediating role of social connectedness and moderating role of exercise self-efficacy
Source: Front Public Health. 2025 Nov 26;13:1701258. doi: 10.3389/fpubh.2025.1701258 (PMC12689978; doi:10.3389/fpubh.2025.1701258)
Supplement: Supplementary file 2 [file Supplementary_file_1.pdf]

Supplement Materials

Table S1. Regression Analysis of the Mediation Model Including Control Variables.

Predictor	Step 1 (Social Connectedness)			Step 2 (Subjective Well-Being)		
	β	SE	t	*β*	SE	t
Age	0.116	0.065	1.791	-0.075	0.051	-1.463
Marital Status	0.004	0.142	0.031	0.086	0.092	0.940
Education Level	-0.012	0.050	-0.228	0.054	0.038	1.425
Monthly Income	0.001	0.086	0.012	0.036	0.066	0.548
Square Dancing	0.585	0.045	12.956***	0.254	0.046	5.531***
Social Connectedness				0.306	0.042	7.255***
R^2		0.307			0.356	
F		34.034***			36.507***	

Note. Supplementary Table S1 presents the regression results when age, marital status, education, and monthly income were entered as control variables. Consistent with the main analysis, the key predictors (square dancing and social connectedness) remained significant, whereas all control variables were non-significant ($p > .05$). Minor numerical differences between this model and the main model (Table 5) are attributable to stochastic variation from bootstrap resampling and do not affect the overall interpretation of the mediation effects.

Table S2. Regression results with standard errors clustered by troupe

Predictor	β	Clustered SE	t	p
Square Dancing → Social Connectedness	0.584	0.050	11.597	<.001
Square Dancing → Subjective Well-Being	0.252	0.063	3.998	<.001
Social Connectedness → Subjective Well-Being	0.300	0.047	6.424	<.001

Note. Regression results estimated with standard errors clustered by troupe (N = 365, 12 troupes).The significance patterns are consistent with the main results in Table 5, indicating that the mediation effects remain robust after accounting for within-troupe correlation.

Reliability and Validity Statistics

Table S3. Construct reliability and convergent validity of the measurement model

Construct	Item	Standardized Loading	Cronbach's α	CR	AVE
Square Dancing	I participated in square dancing on more days per week on average during the past week.	0.718	0.897	0.899	0.529
	I engaged in square dancing very frequently during the past week.	0.696			
	Each of my square dance sessions usually lasts more than 30 minutes.	0.690			
	I am able to maintain participation in square dancing for a relatively long duration each time.	0.767			
	I have been participating in square dancing for many	0.823			

	years.				
	Square dancing has become a long-term leisure activity that I have consistently practiced.	0.734			
	I belong to a regular square dance team and often participate in activities with this team.	0.666			
	I have taken on a specific role in my square dance team (e.g., lead dancer, organizer, or active participant).	0.716			
Social Connectedness	I feel accepted and recognized by others in my square dance team.	0.694	0.870	0.869	0.527
	I feel that I am regarded as a valued member of the team.	0.732			
	I consider myself an indispensable part of the square dance team.	0.707			
	Participating in square dancing gives me a strong sense of group belonging.	0.731			
	I have developed close relationships with members of my square dance team.	0.754			
	I have friends in the team whom I can confide in and rely on.	0.737			
Exercise Self-Efficacy	Even when I feel tired, I am confident that I can keep participating in square dancing.	0.758	0.869	0.869	0.525
	Even when the weather is bad, I am confident that I can keep participating.	0.750			
	Even when my schedule is busy, I can still find time to participate.	0.705			
	Even when I feel slightly unwell, I am confident that I can continue participating.	0.677			
	Even when my friends or family do not accompany me, I can still keep participating.	0.727			
	Even when faced with new or difficult dance moves, I am confident that I can master them through practice.	0.729			
Subjective Well-Being	In most ways, my life is close to my ideal.	0.703	0.917	0.926	0.559
	The conditions of my life are excellent.	0.747			
	I am satisfied with my life.	0.682			
	So far, I have gotten the important things I want in life.	0.721			
	If I could live my life over, I would change almost nothing.	0.708			
	I have felt cheerful and in good spirits.	0.732			
	I have felt calm and relaxed.	0.752			
	I have felt active and vigorous.	0.688			
	I woke up feeling fresh and rested.	0.733			
	My daily life has been filled with things that interest me.	0.726			

Notes:

CR = Composite Reliability; AVE = Average Variance Extracted.

All standardized loadings are significant at $p < .001$.

Recommended thresholds: Cronbach's $\alpha \geq 0.70$, CR ≥ 0.70, AVE ≥ 0.50 (Fornell & Larcker, 1981)

Confirmatory factor analysis (CFA) was conducted using AMOS 26.0. All standardized loadings were significant (λ = 0.666–0.23, p < .001), and standardized residuals were within acceptable limits (|Residual| < 2.0). No items were deleted during the confirmatory factor analysis, as all standardized loadings were statistically significant and above the recommended threshold ($\lambda \geq 0.60$).

Internal consistency was satisfactory (Cronbach's α = 0.869–0.917; CR = 0.869–0.926), and convergent validity was supported (AVE = 0.525–0.559).

The square roots of AVEs exceeded the inter-construct correlations, demonstrating discriminant validity (Fornell–Larcker criterion).

Model Fit Indices for Each Scale

Table S4. Model fit indices for each measurement scale

Scale	χ^2/df	SRMR	RMSEA	GFI	CFI	TLI
Square Dancing Participation	1.689	0.024	0.044	0.976	0.990	0.986
Social Connectedness	1.470	0.019	0.036	0.988	0.995	0.992
Self-Efficacy for Exercise	1.340	0.019	0.031	0.989	0.997	0.994
Subjective Well-Being (Life Satisfaction / WHO-5)	1.470	0.021	0.036	0.975	0.992	0.990

The measurement models of all scales demonstrated satisfactory fit to the data.

For the Square Dancing Participation Scale, χ^2/df = 1.689, SRMR = 0.024, RMSEA = 0.044, GFI = 0.976, CFI = 0.990, TLI = 0.986.

For the Social Connectedness Scale, χ^2/df = 1.470, SRMR = 0.019, RMSEA = 0.036, GFI = 0.988, CFI = 0.995, TLI = 0.992.

For the Self-Efficacy for Exercise Scale, χ^2/df = 1.340, SRMR = 0.019, RMSEA = 0.031, GFI = 0.989, CFI = 0.997, TLI = 0.994.

For the Subjective Well-Being Scale (SWLS and WHO-5 combined), χ^2/df = 1.470, SRMR = 0.021, RMSEA = 0.036, GFI = 0.975, CFI = 0.992, TLI = 0.990.

These indices all fall within the acceptable range (Hu & Bentler, 1999), supporting the structural validity of the adapted Chinese versions.

Confirmatory Factor Analysis of Subjective Well-Being

Table S5. Comparison of One-Factor and Two-Factor Confirmatory Factor Analysis Models for Subjective Well-Being

Model	χ^2/df	CFI	TLI	RMSEA	ΔCFI	ΔRMSEA
One-Factor Model	1.470	0.992	0.990	0.036	0.008	0.036
Two-Factor Model	0.962	1.000	1.001	0.000		

Note: The one-factor model conceptualized subjective well-being as a unified latent construct, while the two-factor model separated cognitive (SWLS) and affective (WHO-5) dimensions. Both models fit well, but the one-factor model was retained for parsimony.
